# Temperature Variation and Host Immunity Regulate Viral Persistence in a Salmonid Host

**DOI:** 10.3390/pathogens10070855

**Published:** 2021-07-07

**Authors:** David J. Páez, Rachel L. Powers, Peng Jia, Natalia Ballesteros, Gael Kurath, Kerry A. Naish, Maureen K. Purcell

**Affiliations:** 1 School of Aquatic and Fishery Sciences, University of Washington, Seattle, WA 98195, USA; knaish@uw.edu; 2 US Geological Survey, Western Fisheries Research Center, Seattle, WA 98115, USA; rpowers@usgs.gov (R.L.P.); jiapeng@sztu.edu.cn (P.J.); naballesterosb@gmail.com (N.B.); gkurath@usgs.gov (G.K.); 3 Shenzhen Customs, Animal & Plant Inspection and Quarantine Technology Center, Shenzhen 518045, China; 4 Quality and Standards Academy, Shenzhen Technology University, Shenzhen 518118, China; 5 Department of Microbiology, University of Alabama at Birmingham, Birmingham, AL 35294, USA

**Keywords:** infectious hematopoietic necrosis virus (IHNV), steelhead trout, salmonid novirhabdovirus, temperature, persistence, immune response

## Abstract

Environmental variation has important effects on host–pathogen interactions, affecting large-scale ecological processes such as the severity and frequency of epidemics. However, less is known about how the environment interacts with host immunity to modulate virus fitness within hosts. Here, we studied the interaction between host immune responses and water temperature on the long-term persistence of a model vertebrate virus, infectious hematopoietic necrosis virus (IHNV) in steelhead trout (*Oncorhynchus mykiss*). We first used cell culture methods to factor out strong host immune responses, allowing us to test the effect of temperature on viral replication. We found that 15 ${}^{\circ }$C water temperature accelerated IHNV replication compared to the colder 10 and 8 ${}^{\circ }$C temperatures. We then conducted in vivo experiments to quantify the effect of 6, 10, and 15 ${}^{\circ }$C water temperatures on IHNV persistence over 8 months. Fish held at 15 and 10 ${}^{\circ }$C were found to have higher prevalence of neutralizing antibodies compared to fish held at 6 ${}^{\circ }$C. We found that IHNV persisted for a shorter time at warmer temperatures and resulted in an overall lower fish mortality compared to colder temperatures. These results support the hypothesis that temperature and host immune responses interact to modulate virus persistence within hosts. When immune responses were minimized (i.e., in vitro) virus replication was higher at warmer temperatures. However, with a full potential for host immune responses (i.e., in vivo experiments) longer virus persistence and higher long-term virulence was favored in colder temperatures. We also found that the viral RNA that persisted at later time points (179 and 270 days post-exposure) was mostly localized in the kidney and spleen tissues. These tissues are composed of hematopoietic cells that are favored targets of the virus. By partitioning the effect of temperature on host and pathogen responses, our results help to better understand environmental drivers of host–pathogen interactions within hosts, providing insights into potential host–pathogen responses to climate change.

## 1. Introduction

Environmental variability has fundamental effects on the evolutionary ecology of host–pathogen interactions [1,2,3,4]. Despite the fundamental role played by environmental variation in the evolutionary ecology of hosts and pathogens, there is still limited empirical knowledge about how environmental variation affects within-host infection processes, particularly whether infection outcomes under different environmental conditions result from effects of the environment on the host, the pathogen or on both interacting organisms. Environmental parameters such as water quality and temperature may have a particularly profound effect on the infection processes of aquatic poikilothermic animals [5]. Warming water temperatures projected under climate change scenarios may facilitate invasion of certain pathogens or cause more severe disease outbreaks in aquatic populations [6]. Environmental variability may alter other aspects of host–pathogen dynamics such as driving a switch between acute and chronic infection manifestations [7].

*Salmonid novirhabdovirus* (syn. infectious hematopoietic necrosis virus; IHNV) is an acute viral pathogen of wild and cultured salmon and trout populations in North America, Asia, Europe, and The Middle East [8,9]. IHNV has a negative-sense, single-stranded RNA genome and this virus groups within the family Rhabdoviridae and genus *Novirhabdovirus*. There are five major genogroups of IHNV worldwide, three of which (U, M, and L genogroups) circulate in western North America [10]. The virus is endemic and epidemic in many hatchery-based breeding programs that seek to conserve salmonid populations in western North America [9]. The disease, infectious hematopoietic necrosis (IHN), is associated with widespread degenerative necrosis in hematopoietic tissues and other organs, often leading to rapid onset of mortality in juvenile fish. However, other IHN disease manifestations have been described in rainbow trout (*Oncorhynchus mykiss*) that are associated with skin (epitheliotropic) and brain (neurotropic) infections [11]. In the acute hematopoietic form, high mortality occurs, but survivors typically clear the virus and develop broadly protective immunity to re-infection [12,13,14]. Chronic brain-associated infections have been linked to cooler temperatures in sockeye salmon (O. nerka) [15], but the varied disease manifestations occur in farmed rainbow trout held in spring-fed water with a constant temperature of approximately 15 ${}^{\circ }$C. Thus, the relative importance of host, pathogen or environmental factors that lead to these alternative disease manifestations are not defined.

IHNV is considered a cold-water adapted pathogen with acute epidemics observed when water temperature ranges from 8 to 15 ${}^{\circ }$C [16]. Studies of a related *Novirhabdovirus*, viral hemorrhagic septicemia virus (VHSV), suggest that colder water temperatures exacerbate fish mortality and lead to persistent chronic infections [17,18]. However, mixed results have been obtained for the relation between water temperature and host mortality from IHN disease. Colder water temperatures have been associated with lower overall mortality in some studies [19,20] but not necessarily with all IHNV strains [21]. Additionally, prior studies of IHNV have obtained mixed results as to whether viral persistence occurs and whether it varies with water temperature. For example, some studies demonstrate long-term persistent infections [15,22,23,24,25,26], while other studies did not find support for persistent infections [27,28]. Many factors varied among these prior studies, but the totality of the findings support the hypothesis that water temperature not only affects overall mortality, but also the within-host processes of replication and clearance. Persistently infected hosts that do not fully clear the virus may be a mechanism to maintain the virus in the host population, particularly because shed IHNV is dispersed and decays in the natural environment [29]. Thus, within-host persistence is an important IHNV fitness trait that may be modulated by environmental conditions.

In this study, we sought to characterize how three different environmental temperatures influenced in vivo clearance or persistence of a field isolate of IHNV in its natural host, steelhead trout (*Oncorhynchus mykiss*). From 2007–2011, a variant of IHNV classified in the M genogroup—subgroup D (or MD type) emerged on the Washington Coast (WA, USA) causing dramatic mortality in coastal steelhead trout populations and prompting aggressive eradication actions [30]. We used a field isolate of IHNV from this emergence event to first assess the temperature dependent replication of the virus in a fish cell line, where the host response is minimized. Next, we conducted in vivo IHNV exposure studies to evaluate the effect of three water temperatures (6, 10, and 15 ${}^{\circ }$C) on infection levels, virus clearance/persistence and humoral immune responses over an 8-month period. Finally, we identified which host tissues were associated with persistent IHNV RNA. Our results support the hypothesis that MD IHNV replicates faster at warmer temperatures, but the long-term persistence in steelhead increases at colder temperatures. The frequency of persistent infection was associated with slower virus decay rate and/or reduced expression of neutralizing antibodies. Persistent virus was most frequently localized to the kidney and/or spleen. Our results highlight the importance of water temperature as an important parameter for models seeking to understand the transmission of IHNV.

## 2. Materials and Methods

### 2.1. Virus and Cell Culture

The Qts07 IHNV strain (belonging to genogroup MD; genotype mG110M) from a diseased steelhead in 2007 [30] was propagated in a Fathead minnow cell line (*epithelioma papulosum cyprini*; EPC) at 15 ${}^{\circ }$C. The cells were cultivated in minimal essential medium (MEM; Gibco) supplemented with 10% fetal bovine serum (HyClone, Thermofisher Scientific, Waltham, MA, USA). The final viral supernatant stocks were quantified by standard plaque assay [31] and stored at −80 ${}^{\circ }$C until used.

### 2.2. Viral Replication in Cell Culture at Different Temperatures

Viral growth in EPC cells was compared at three temperatures, 8, 10 and 15 ${}^{\circ }$C (it is impossible to culture EPC cells below 8 ${}^{\circ }$C). Cells were seeded into 24-well plates and, after a 24-h incubation, the confluent monolayers of EPC cells were infected with 0.3 mL of virus at a multiplicity of infection (MOI) of 0.1 and 0.01 plaque forming unit (PFU) per cell (two duplicate wells per treatment condition). MOI is a measure of host cell availability such that a MOI of 0.1 and 0.01 represents, on average, a ratio of 10 and 100 host cells per infectious viral unit, respectively. At the end of a 1-h absorption period at 15 ${}^{\circ }$C, the inoculum was removed and 1 mL of MEM containing 5% fetal bovine serum was added to each well, and the plates were incubated at their respective treatment temperatures. At each time point, 200 μL of cell culture supernatant was sampled from the duplicate wells at 1, 2, 3, 5, 7, and 10 days post inoculation (dpi) and stored at −80 ${}^{\circ }$C. Each temperature treatment was repeated three times. Virus titers at each time point were determined by plaque assay using duplicate technical replicates.

Linear mixed effects models were used to determine whether viral replication in cell culture (measured as the log10 viral titer of the PFU/mL) depended on incubation temperature, the time since inoculation, and host cell availability (i.e., by varying MOI), while controlling for variation across replicates. Preliminary data exploration showed evidence of a non-linear relationship between viral replication and time. Therefore, viral replication was modeled as a function of linear and quadratic effects of the time since inoculation (measured in days), incubation temperature and MOI treatment, which were fit as fixed effects. We also included pairwise interaction terms and the triple interaction term between these explanatory variables. In this model, the random effects were defined by the three replicated samples collected at each sampling time from independent experiments and by the two technical replicates associated with plaque testing wells. Using this model as the most complex model, we then constructed 16 additional candidate models that omitted one or more of the fixed effects, such as the triple and double interaction terms. We then used Akaike information criteria (AIC) and Akaike weights (wAIC, which quantifies the probability that each model is the most correct model given the data and the candidate models) for model selection [32]. Because our models include both a linear and a quadratic effect of the time since inoculation, the effect of this variable was tested by comparing AIC scores with a simpler model that omitted both the linear and quadratic effects (i.e., because omitting the linear effect but including the quadratic effect does not make biological sense).

### 2.3. Steelhead Survival after Virus Exposure at Different Temperatures

All animal experiments were approved by the University of Washington Institutional Care and Use Committee under protocol 3042-15. Eyed steelhead trout eggs were obtained from Quinault National Fish Hatchery, Humptulips, WA, USA (brood year 2013) and reared to approximately 8 g on sand-filtered, UV-treated freshwater at the U.S. Geological Survey (WA, USA). Fish were randomly assigned to treatment groups and acclimated to 6, 10, or 15 ${}^{\circ }$C for 7 days prior to virus exposure. For each temperature treatment group, one tank containing 400 fish was challenged with 5 × 10${}^{4}$ PFU/mL of IHNV by static immersion for 1 h. This viral dose was shown to cause approximately 30–40% mortality in pilot studies (data not shown). A second tank for each temperature treatment containing 200 fish was mock challenged with medium alone, forming control groups. Tanks were monitored daily for mortalities or severely moribund fish; moribund fish were humanely euthanized with an overdose of buffered tricaine methanesulfonate (MS222, Syndel). Due to a technical issue that occurred in the tank of challenged fish in the 15 ${}^{\circ }$C treatment at 95 days post exposure (dpe), we were forced to halt data collection for this treatment, so both the 15 ${}^{\circ }$C control group and the virus-challenged fish were euthanized. For the 6 and 10 ${}^{\circ }$C treatment groups fish in both the control and virus-exposed tanks were monitored through 270 dpe. All mortalities, moribund fish that occurred after 30 dpe, and the survivors at 270 dpe from the IHNV challenged groups (15 fish at 6 ${}^{\circ }$C and 7 fish at 10 ${}^{\circ }$C) were tested for viable virus using plaque assay method for fish tissues [31].

To estimate survival probabilities across temperature treatments, individuals were classified as either succumbed to infection or as surviving to 95 dpe (15 ${}^{\circ }$C group) or 270 dpe (6 and 10 ${}^{\circ }$C groups). We used survival analyses which allowed to estimate the daily likelihood of death given that the sample had survived so far (this likelihood is also known as the event hazard). Because of evidence of non-proportional hazards, we used a weighted cox regression with right censoring [33]. We used right censoring because we did not know whether disease-related mortality would have occurred in fish that were sampled or euthanized over the course of the experiment or after the experiment termination date. Thus, a fish’s event time (i.e., time to death) and event status (i.e., dead or censored) were modeled as a function of the temperature treatment (i.e., 6, 10, and 15 ${}^{\circ }$C). We excluded control fish from this model because the observed mortality in control groups was verified to be unrelated to IHN disease (i.e., all control samples tested negative for IHNV). Hazard ratios were then estimated for the risk of fish death with increasing temperature. For these survival analyses, we used the survival [34] and coxphw [35] packages available in R [36].

### 2.4. Viral Decay in Steelhead Exposed to Virus at Three Temperatures

Steelhead exposed to IHNV at three temperatures (as described above) were sampled at 7, 17, 31, 55, and 91 dpe; the 6 and 10 ${}^{\circ }$C groups were also sampled at 179 and 270 dpe. We sampled a larger number of individuals at later time points to obtain a more precise estimate of the infection prevalence, which was expected to be low with increasing dpe. Specifically, for virus-exposed groups, six individuals were sampled at 7 and 17 dpe, whereas 28–30 individuals were sampled for all time points thereafter. For the control groups, six individuals were sampled per temperature treatment from all time points. For all samples, fish blood was collected using Natelson tubes (Thermo Fisher Scientific) and kept on ice during sampling, then transferred to 4 ${}^{\circ }$C to clot overnight. Blood samples were subjected to centrifugation at 12,000× *g* for 10 min, and serum was preserved in a new tube at −80 ${}^{\circ }$C until used. For each fish, small sections of pelvic fin, gill, anterior and posterior kidney, spleen and brain were pooled (total tissue weight approximately 50 mg) and stabilized in RNAlater following manufacturer’s instructions (Invitrogen, Waltham, MA, USA). A second tissue pool was collected in RNAlater for the purpose of extracting RNA from individual tissues from fish that tested positive for persistent IHNV RNA. All surviving fish were euthanized with MS222 at 270 dpe and whole fish were frozen at −80 ${}^{\circ }$C for plaque assay analysis.

A pooled RNA tissue sample collected from each fish was processed to determine the presence or absence of IHNV RNA. Tissue samples were homogenized with a Fast-Prep^TM^ 24-bead beater (MP Biomedical) for 20 s, using 1 mm of zirconia/silica beads (BioSpec Products, Inc.) and Buffer RLT (Qiagen, Hilden, Germany). Total RNA was extracted using the RNeasy Mini-kit (Qiagen), following the manufacturer’s protocol. Total RNA was eluted in 60 mL of nuclease-free water, quantified using the Nanodrop ND-1000 (Thermo Scientific) and stored at −80 ${}^{\circ }$C until used. Total RNA was quantified using the Nanodrop ND-1000 (Thermo Scientific). Reverse transcription was initiated with 1.5 μg of total RNA in a reaction volume of 20 μL (ABI High Capacity cDNA kit, Thermo Fisher), and cDNA was diluted 1:5 with nuclease-free water. RT-rPCR was performed using the IHNV universal N-gene assay and methods developed by Purcell et al. [37]. An artificial positive control plasmid (plasmid encoding the RT-rPCR target region; IDTDNA Inc.) was used to quantify copies of the IHNV N gene per μg of total RNA. To estimate viral decay rates, we used

(1) $$y\left(t\right)=L+ae^{-\lambda t},$$

to quantify how long virus persisted within hosts across the temperature treatments. Here, *y* is the viral load (number of RNA copies/μg RNA measured on a log10 scale), *L* is a minimum saturating parameter, *a* is indicative of the number of RNA copies present at time 0 (which is given by L+a), λ is the decay rate parameter (in day${}^{-1}$ units) and *t* is time (in days). This model, (eqn 1) was fit to the data using a non-linear least-square method, implemented in the nlme package in R [38]. Our comparisons across temperature treatments especially focus on the estimates of the saturating *L* and decay λ parameters, because these values indicate viral load values at which the pathogen persists and how fast this value is reached, respectively. We also used the viral load value at the time of half the saturating value to compare the effect of viral load decay between temperature treatments. Such half-time (HT) was calculated as $HT=-\frac{log(\frac{a-L}{2a})}{\lambda }$.

### 2.5. Individual Steelhead Tissues Harboring Long-Term Persistent IHNV RNA

For all steelhead testing positive for IHNV on days 179 and 270 dpe (6 and 10 ${}^{\circ }$C groups), we examined whether long-term persistence was tissue specific. Kidney/spleen, brain, gill and fin tissues were identified from the second tissue pool and these individual tissues were extracted and subjected to IHNV RNA detection, as described above.

To quantify tissue-specific viral persistence, we used a logistic regression model, where the presence of virus was modeled as a function of the interaction between the tissue type, dpe, and the rearing water temperature (6 and 10 ${}^{\circ }$C). We then used model selection procedures, as described above to identify the most parsimonious model given the data.

To determine whether infection in one tissue depended on the infection status of other tissues we categorized tissue infection into whether IHNV was detected in (1) the kidney/spleen only, (2) other tissues only, and (3) in the kidney/spleen and other tissues. This categorization was necessary because our data did not contain enough positive samples to compare pairwise probabilities of infection between all tissues (see Supporting Material, Figure S1). First, we tested whether kidney/spleen infection depended on the infection status of other tissues, temperature, and dpe. We then tested whether infection in other tissues depended on the infection status of the kidney/spleen and the other variables. To compare individual viral loads between tissues, we used linear models to determine (1) if the average viral loads in the kidney/spleen were different from the average viral load in other tissues given that both the kidney/spleen and other tissues were infected and (2) if the average viral load in the kidney/spleen was different from the average viral load in other tissues given that both types of tissue were solely infected.

### 2.6. Steelhead Specific Immune Response to IHNV

The steelhead specific immune response was measured using a complement-dependent neutralizing antibody assay modified from previous studies [39,40]. Serum samples from steelhead exposed to IHNV at three temperatures were heat inactivated for 30 min at 45 ${}^{\circ }$C to inactivate complement. A varying number of fish sera were analyzed (sample N ranged 15–30). The serum samples were serially diluted in 2-fold increments from 1:20 to 1:2560 with MEM-0. Equal parts diluted serum and virus at 3 ×$10^{3}$ PFU/mL (total volume 30 μL) were mixed briefly and incubated at 15 ${}^{\circ }$C for 30 min. A 1:20 dilution of complement serum (pooled serum collected from naïve rainbow trout) was added (15 μL) and incubated at 15 ${}^{\circ }$C for 1 h. The same procedure was followed for negative controls (MEM instead of serum). EPC cells were seeded on 96-well cell culture treated sterile plates approximately 24-h before the assay. A serum–virus mixture was inoculated onto confluent cells, pre-treated with 20 μL of 7% polyethylene glycol (PEG). Each sample was inoculated into triplicate wells. Nine wells of the negative control were included per day. After a 30-min adsorption period at room temperature, 100 μL methylcellulose overlay was added [31], and the plates were held at 15 ${}^{\circ }$C for 7 days. After 7 days, the plates were stained with crystal violet and the plaques were counted. The plaque counts of the negative control wells were averaged over the entire experiment (N =21 runs). Samples with less than or equal to 50% of the number of plaques in negative serum controls at the 1:80 dilution (as well as the 1:20 and 1:40 dilutions) were scored as positive for antibody. The 1:80 dilution was selected to minimize the risk of detecting false positives in the 1:20 or 1:40 dilutions. Samples that were positive for the presence of neutralizing antibodies were categorized as having small (positive at 1:80 or 1:160), intermediate (1:320 or 1:640) or large (1:1280 or 1:2560) titers.

To test if the host antibody response depended on the time since virus exposure and temperature treatments, we used a logistic regression model. Antibody detection was a function of linear and quadratic effects of temperature treatment and dpe. We then built simpler models and used AIC scores to select the model with highest explanatory power.

## 3. Results

### 3.1. Temperature-Dependent Virus Replication in Cell Culture

**Figure 1:**
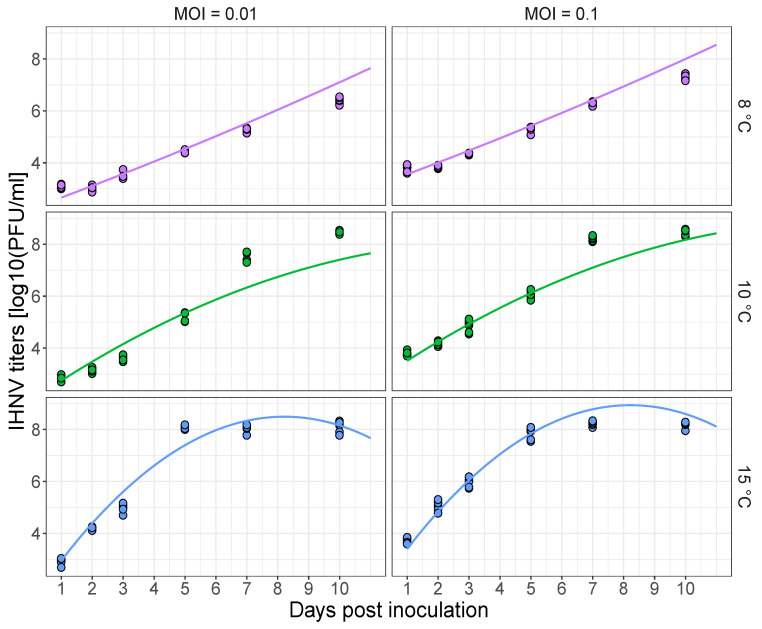
Infectious hematopoietic necrosis (IHNV) titer [log10 (plaque forming units (PFU)/mL)] in EPC fish cells as a function of the time since inoculation for the different incubation temperatures in ${}^{\circ }$C and multiplicity of infection (MOI) ratios. Lines are the best fit of a linear mixed model. The dots are the PFU data across different temperatures (colors) and MOI treatments (left and right columns).

We found that increasing temperature resulted in faster viral replication and an earlier saturation of viral loads (e.g., comparing the viral loads for a given dpi within MOI treatments, Figure 1). For instance, viral load plateaued in the 15 ${}^{\circ }$C group at approximately 8 log10 PFU/mL between 5 and 7 dpi, whereas viral loads were still increasing at these time points in the 8 and 10 ${}^{\circ }$C groups. This saturation is likely caused by near complete cytopathic effect with most host cells dead. A significant effect of initial host cell availability was also supported by the interaction between temperature and MOI (*w*AIC for best Model = 0.60, Table S1). Taken together, higher viral loads were achieved with increased host cell availability and increased temperature, with earlier viral load saturation at the highest temperature.

### 3.2. Survival of Steelhead Following Virus Exposure at Three Temperatures

**Figure 2:**
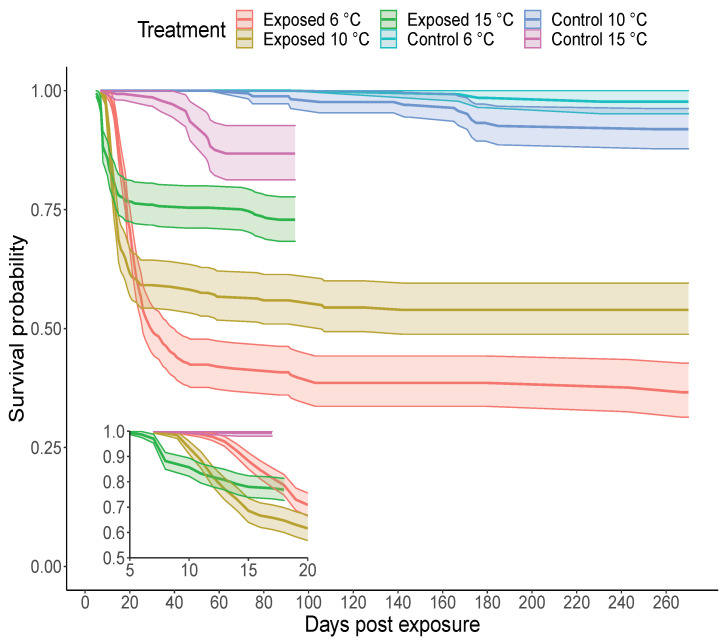
Kaplan Meier plot of the survival probability as a function of time for the different group/temperature treatment combinations. Steelhead were exposed to either infectious hematopoietic necrosis virus (IHNV) or buffer alone (control) at three temperatures. Shaded areas around the solid lines are 95% confidence intervals of survival probabilities. The main plot shows survival probabilities over the 270-day experiment. The 15 ${}^{\circ }$C temperature groups were terminated at 95 days post exposure (see methods). The inset panel provides better resolution of the onset of mortality over the first 20 days in the IHNV exposed steelhead.

The onset of mortality in IHNV exposed fish was at 9, 7, and 5 dpe in the 6, 10 and 15 ${}^{\circ }$C temperature groups, respectively, (Figure 2, insert). The plateau in IHNV mortality occurred earlier in the 15 ${}^{\circ }$C group (∼15 dpe) relative to the 10 ${}^{\circ }$C (∼25 dpe) and 6 ${}^{\circ }$C (∼40 dpe) groups. After plateau, fish exposed to IHNV and held at the 15 ${}^{\circ }$C treatment had an overall lower mortality than virus exposed fish held at colder temperatures. Specifically, survival analyses suggest that increasing water temperature decreased fish mortality by a factor of 0.75 (with confidence intervals, CI = 0.68, 0.85). At 95 dpe, the cumulative percent mortality (CPM) was 55.8%, 39.5% and 24.0% in the 6, 10 and 15 ${}^{\circ }$C temperature groups, respectively; the 15 ${}^{\circ }$C treatment was terminated at this time point. Only negligible mortality occurred after 95 dpe in the remaining two temperature groups. The final CPM at 270 dpe was 56.5% and 40.8% at 6 and 10 ${}^{\circ }$C, respectively.

The low level of mortality in all control groups (2%, 6.6% and 12% mortality at 6, 10 and 15 ${}^{\circ }$C, respectively, Figure 2), was confirmed to be non-specific as these control mortalities were negative for IHNV by plaque assay. For the IHNV exposed groups after 30 dpe, viable virus was isolated from 87.5% (14/16) and 90% (9/10) mortalities in the 6 and 10 ${}^{\circ }$C groups, respectively. These virus positive mortalities were observed from 43 to 267 dpe with titers ranging from 7.3 ×$10^{2}$ to 2.6 ×$10^{6}$ PFU/g. A random sample of 270 dpe survivors did not test positive for viable virus but the sample sizes tested were small (N=15 for the 6 ${}^{\circ }$C; N=7 for 10 ${}^{\circ }$C group).

### 3.3. Viral Decay in Steelhead Exposed to Virus at Three Temperatures

**Figure 3:**
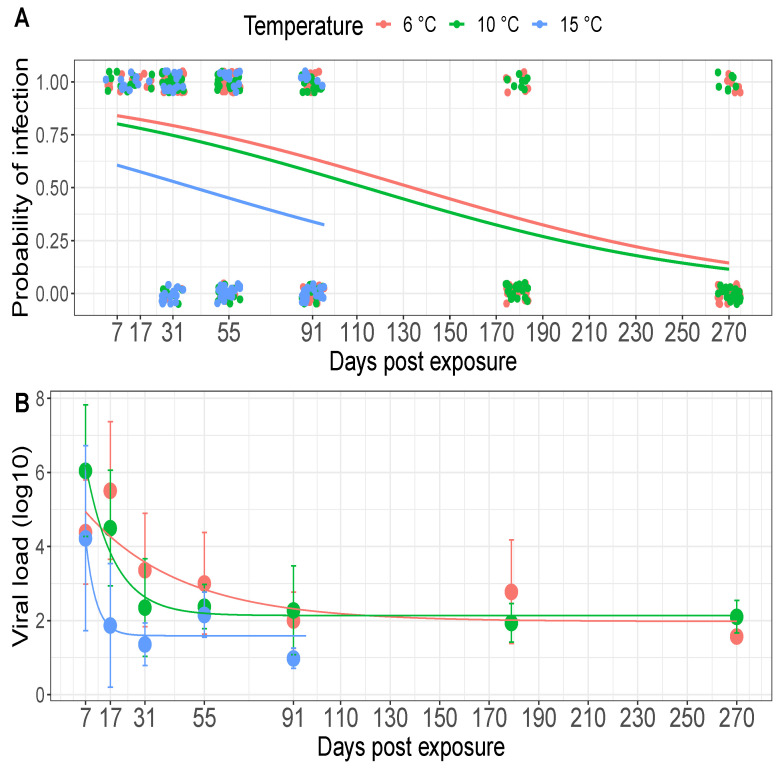
(**A**) Probability of infection of juvenile steelhead over time across temperature treatments. Lines are the best fit of a logistic regression model, whereas the dots are the data, with samples testing positive (y-value =1) and negative (y-value =0) for viral RNA. Dots were slightly shifted on the horizontal and vertical axes for visibility. (**B**) Viral load decay in virus-positive fish over time. Lines represent the fit of the viral decay model (eqn 1). Large dots are the mean data values and the error bars extend ±1 standard deviation around the mean. The 15 ${}^{\circ }$C group was terminated at 95 days post exposure (dpe) while the 6 and 10 ${}^{\circ }$C groups were terminated at 270 dpe.

No tissue pools (fin, gill, kidney, spleen and brain) from control fish tested positive for IHNV RNA with the PCR tests. Pooled tissue samples from IHNV exposed fish testing positive for viral RNA was interpreted as evidence of viral infection. A 100% prevalence of viral infection (out of 6 samples) was observed at 7 dpe in all three temperature groups and the prevalence declined at subsequent time points (Figure 3A). At 91 dpe the proportions of fish with detectable viral infection were 50% (15/30), 50% (15/30), and 25% (7/28) at 6, 10, and 15 ${}^{\circ }$C, respectively. By the end of the study, at 270 dpe there were still 23% (7/30) positive fish in each of the 6 and 10 ${}^{\circ }$C treatments.

The probability of viral infection depended on the dpe and the temperature treatments. As dpe increased, the probability of infection decreased, reaching levels below 50% after approximately 45 dpe in the 15 ${}^{\circ }$C group and 125 dpe in the 6 and 10 ${}^{\circ }$C treatments. The probability of viral infection was also lower in the 15 ${}^{\circ }$C compared to the 10 and 6 ${}^{\circ }$C treatments (Figure 3A). The probability of infection in the 10 ${}^{\circ }$C treatment was intermediate to the 6 and 15 ${}^{\circ }$C treatments but was indistinguishable to the 6 ${}^{\circ }$C treatment (Figure 3A). Based on AIC scores, the greatest support was found by a model containing the interaction between temperature and the time since exposure (*w*AIC =0.64; see full AIC table in the supporting information Table S2), with weaker support for a model omitting this interaction (*w*AIC =0.36). Thus, while this interaction likely explains the differences in the probability of infection between the 15 ${}^{\circ }$C and both the 6 and 10 ${}^{\circ }$C treatments, the interaction is only weakly informative.

The decay model indicated that the viral load decayed at a slower rate and had a larger half-time in the 6 ${}^{\circ }$C temperature treatment compared to the 10 ${}^{\circ }$C treatment (Figure 3B, λ estimate in Table 1), suggesting that virus can persist for longer within hosts at low temperature. Consistent with this trend, the decay rate estimate was larger in the 15 ${}^{\circ }$C treatment compared to the 10 and 6 ${}^{\circ }$C treatments, however, 95% confidence intervals around the 15 ${}^{\circ }$C estimate overlapped 0 (Table 1), making it difficult to draw strong conclusions about differences between the 15 ${}^{\circ }$C and the other two treatments (Table 1).

### 3.4. Specific Steelhead Tissues Harboring IHNV RNA

**Figure 4:**
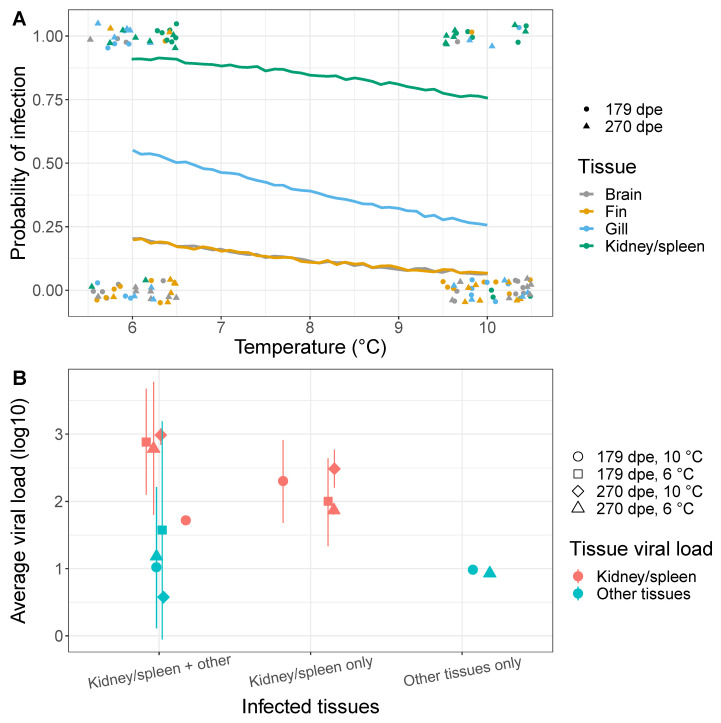
Steelhead tissues harboring persistent IHNV RNA at 179 and 270 dpe. (**A**) Probability of infection in different tissues as a function of temperature (6 or 10 ${}^{\circ }$C). Point symbols are the data, which were slightly shifted to improve visualization. Positive tests are along y =1, whereas negative tests are along y =0. The lines are the fit of a logistic regression model (and they have been slightly rugged to distinguish the line for the fin and brain tissues because the brain and fin had the same number of infected samples and so their probabilities of infection were identical). (**B**) Viral loads in kidney/spleen tissues or in other tissues (brain, fin, or gill). Each dot corresponds to the average viral load when infection was in the kidney/spleen alone, the kidney/spleen and other tissues, and other tissues alone. The viral load for other tissues is the average of the viral loads across all non-kidney/spleen tissues. Thus, if all tissues were infected, the viral load for “other tissues” was calculated by averaging the viral loads found in the brain, gill and fin. However, if only the gill and kidney/spleen were infected, the viral load for “other tissues” was the viral load measured for the gill. Error bars (±standard deviation) are shown for groups containing more than one observation (see Figure S1).

Fish which tested positive for IHNV using pooled internal and external tissues at later time points (179 and 270 dpe) were subjected to further testing by using a second set of tissue samples to determine which specific tissues were associated with persistent viral infection. We found positive fish had some tissues testing positive and other tissues testing negative for viral RNA, and that positive tissues were principally the kidney/spleen (Figure 4A). Because of limited sample sizes we could not draw meaningful conclusions about non-kidney, tissue-specific differences in infection (Figure S1). Specifically, at 10 ${}^{\circ }$C, only 1 fish was positive for IHNV in the brain. Similarly, at 6 ${}^{\circ }$C only one fish tested positive in non-kidney/spleen tissues. We therefore pooled all non-kidney tissues together to draw meaningful inferences for the tissue positivity rate (i.e., between kidney/spleen and other tissues).

By comparing fish reared in the 6 and 10 ${}^{\circ }$C temperatures, we found that increased rearing temperature reduced the prevalence of viral RNA across all tissues, with this reduction being larger in the gill tissue (Figure 4A). Indeed, our model selection procedure suggested that tissue viral prevalence was best explained by the effects of temperature and the tissue identity (*w*AIC =0.54), with a limited role of time (i.e., 179 and 270 dpe, see Table S3 for full AIC scores).

The viral load in the kidney/spleen was consistently higher than the viral load in other tissues. This was observed when comparing viral loads from fish with infections only in the kidney/spleen vs. those with infection only in other tissues (ΔAIC =8.63, Figure 4B). Higher viral loads in the kidney/spleen compared to other tissues was also observed when infection occurred jointly in the same fish (ΔAIC =9.97; Figure 4B).

### 3.5. Steelhead Specific Immune Response to IHNV

**Figure 5:**
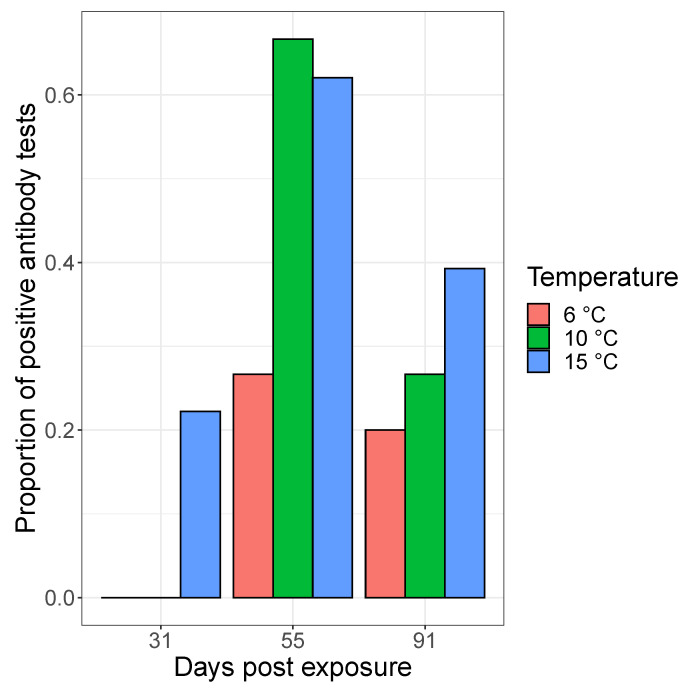
Proportion of steelhead with detectable neutralizing antibodies to IHNV as a function of time and temperature treatments.

In fish serum samples collected at 31, 55, and 91 dpe, IHNV neutralizing antibodies were detected at a higher frequency in fish held at 10 and 15 ${}^{\circ }$C temperatures compared to fish held at 6 ${}^{\circ }$C (Figure 5; Table 2). Across all temperature treatments, the proportion of samples testing positive for antibodies peaked at 55 dpe (Figure 5). This was confirmed by our model selection results, which showed the greatest support for a model containing a quadratic effect of dpe (explaining the peak of neutralizing antibodies at 55 dpe) and temperature (*w*AIC =0.73). A model containing the interaction between temperature and time since exposure also had some support (*w*AIC =0.27). Fish held at the 15 ${}^{\circ }$C treatment produced antibodies sooner than the 10 and 6 ${}^{\circ }$C treatment groups, with 4 samples testing positive at 31 dpe at the conservative dilution threshold of 1:80 (Figure 5, Table 2).

Our results further suggest that the magnitude of the antibody response varies with dpe and the temperature treatments (Table 2). Positive samples were categorized into three categories to define the magnitude of response that ranged from small (positive at a 1:80 or 1:160 dilution), intermediate (positive at a 1:320 or 1:640 dilution) and large (positive at a 1:1280 or 1:2560 dilution). More fish in the 10 and 15 ${}^{\circ }$C treatment groups had antibody responses classified as intermediate or large compared to the 6 ${}^{\circ }$C treatment. For instance, 20% of the 10 ${}^{\circ }$C fish and 14% of the 15 ${}^{\circ }$C fish had an antibody response classified as large compared to only 3% of the 6 ${}^{\circ }$C fish at the 55 dpe time point.

## 4. Discussion

For many host–pathogen interactions, warmer temperatures are associated with increased disease prevalence, including other diseases in salmonids and other fish species [41], as well as other vertebrates [42]. We designed our experiments to test for differences in IHNV replication at or below the optimal viral replication temperature, which is approximately 15 ${}^{\circ }$C [19,43].

A comparison of our results to other IHNV-salmonid studies suggests that host–pathogen-temperature interactions may differ depending on the specific host–pathogen combination. For example, early studies in hatchery sockeye salmon (*O. nerka*) with IHNV (assumed U genogroup) observed lower mortality in fish held at 20 ${}^{\circ }$C, which is above the optimal virus replication temperature, compared to fish held at 12 ${}^{\circ }$C [19]. In more recent studies, low mortality at 15 ${}^{\circ }$C relative to 10 ${}^{\circ }$C was observed for sockeye salmon exposed to some IHNV strains [21]. By contrast, significantly greater mortality at 15 ${}^{\circ }$C relative to 10 ${}^{\circ }$C was observed for one of two M virus strains (MB subgroup) in a commercial stock of rainbow trout [21]. This is consistent with an early hypothesis by Amend and Smith [44] suggesting that adaptation to the constant 15 ${}^{\circ }$C water temperatures in southern Idaho aquaculture was a component of the evolution of IHNV in rainbow trout that resulted in the M genogroup lineage. The MD subgroup lineage utilized herein was first recorded in Idaho trout farms [45], followed by detection in Columbia River Basin steelhead populations in the 1990s [46]. Our specific MD IHNV isolate was derived from hatchery steelhead outbreaks on the Washington Coast in 2007 [30]. In our study, steelhead infected with this isolate suffered lower mortality at 15 ${}^{\circ }$C than at 10 ${}^{\circ }$C, which is in contrast to the results using the MB subgroup strain in rainbow trout [21]. However, both studies observed a more rapid onset of mortality at 15 ${}^{\circ }$C. In studies of the related VHS virus, it has been shown that the polymerase protein (encoded by the L gene) is responsible for differences in temperature sensitivity [47]. The L genes of these two VHSV strains differed by 31 amino acids but the exact amino acid(s) responsible for the phenotypic change was not identified. For IHNV, the L gene of the MD and MB strains used herein and by [21] differed by 28 amino acids, suggesting that viral genetic variation may also influence temperature-dependent growth. Thus, the variable effect of IHNV isolate, and possible differences in rainbow versus steelhead trout hosts, further highlight the need to consider pathogen evolutionary history in addition to environmental variables when studying disease drivers in salmonid populations.

We show that parsing out the effects of temperature on the host and pathogen provides insights into mechanisms driving variation in infection outcomes. The observation that colder temperatures (6 ${}^{\circ }$C) led to slower IHNV decay and higher mortality compared to warmer temperatures (10 and 15 ${}^{\circ }$C) may be a property associated with cold-water adapted pathogens. Clinical disease caused by several other salmon pathogens, including the bacteria *Renibacterium salmoninarum* and *Flavobacterium pyschrophilum*, are known to be exacerbated at colder temperatures [48,49,50]. Colder water is linked to high mortality and fish kills events of marine fish with VSHV [51]. These pathogens show a similar characteristic to our findings.

In cell cultures, these pathogens replicate more rapidly at warmer, compared to colder temperatures [52,53,54], but they cause higher host mortality at colder temperatures in vivo. Our statistical results on the cell culture experiments further suggest that viral replication is principally influenced by the effects of temperature and host-cell availability rather than by effects of time since inoculation and point to a larger effect of temperature on the host immune response relative to the effect on the pathogen life cycle. The early innate immune response is essential for naïve fish to survive a first exposure to acute IHNV infection [14]. Here, the half-life of IHNV at 15 ${}^{\circ }$C was estimated as 3 dpe (with high uncertainty). At these early time points, any immune response would certainly be mediated by innate effector systems, such as the type I interferon (IFN) pathway.

In prior studies, rainbow trout exposed to an IHNV MB strain at 15 ${}^{\circ }$C mounted a peak innate IFN response at 3 dpe [55,56]. In the present study, our first timepoint at 7 dpe was likely too late to capture the early dynamics of the innate IFN response. Using evidence of increased production of neutralizing antibodies at 15 ${}^{\circ }$C compared to 10 and 6 ${}^{\circ }$C, we hypothesize that increased IHNV clearance at warmer temperatures is due to a more rapid and/or effective specific immune response that quickly suppressed effects of pathogen replication. Our results from the in vivo experiments support this hypothesis because the measured viral loads were lower in the 15 ${}^{\circ }$C treatment compared to the other temperature treatments throughout the experiment. Furthermore, Lorenzen et al. [57] found that colder temperatures delayed, and prolonged the innate immune responses and depressed the specific antibody response in rainbow trout following vaccination, which is also consistent with our results. Our results further showed that fish held at 10 ${}^{\circ }$C also increased antibody production within the first two months after the challenge and that the overall prevalence of neutralizing antibodies was greater at 10 vs. 6 ${}^{\circ }$C. This is consistent with the observations of a shorter IHNV half-life and lower cumulative mortality at 10 vs. 6 ${}^{\circ }$C. However, it is interesting to note that these two temperature groups had similar probabilities of infection and low viral loads at the final time point. We evaluated neutralizing antibody response, but other temperature-sensitive immune mechanisms such as cellular mediators may also be important for clearing the virus [58,59] and could complement our conclusions.

Studies in commercially cultured rainbow trout described hematopoietic (kidney/spleen), epitheliotropic (skin) and neurotropic (brain) IHN manifestations [11]. We observed that long-term persistence was more likely to occur within kidney/spleen tissues as both the probability of infection in this organ and virus titers of infected individuals was higher over time relative to the other tissues (brain, fin and gill). In teleost fishes, the kidney is a key organ involved in mounting immune responses against a variety of pathogens [60]. Thus, failure to clear virus from the kidney may actually contribute to the development of long-lasting immunity as the presence of IHNV in this tissue may stimulate continuous antibody production and greater avidity by increased specificity of the adaptive immune response to specific pathogen types [61]. From the pathogen’s perspective, however, persistence in the kidney may be an optimal strategy to persist within the host, as structures within the kidney are key producers of hematopoietic cells, which IHN exploits. Additionally, infecting kidney tissues may favor shedding and between-host transmission by having close access to the host’s excretion functions. Additional work is needed to determine if the host tissue associated with long-term persistence of IHNV is consistent across all IHNV genogroups and hosts. For instance, sockeye salmon infected with the U genogroup had persistent virus in the brain but not the kidney at 9 months post-exposure [15]. Future work geared towards understanding the link between long-term virus persistence in the kidney and virus shedding will likely inform us on epidemiological costs of building long-term protective immunity and on the mechanisms through which within-host infection processes translate to between-host dynamics.

The ability of IHNV to persist long-term in surviving fish has been of interest for many decades because this may be a mechanism for maintenance of the virus in salmon populations [9]. An acute IHNV infection typically leads to either mortality or recovery with viral clearance in most fish [27,28]. However, a small number of previous studies have reported that some fish carry persistent RNA over the long-term [15,23,24]. These studies used PCR-based methods that have higher sensitivity than cell culture. Using these methods, the frequencies of viral persistence we report here are substantially higher than we anticipated, with evidence of viral infection in half of the fish at 45 dpe at 15 ${}^{\circ }$C, and 125 dpe at 6 or 10 ${}^{\circ }$C. Even at the longest time tested, 270 dpe, we detected infection in 7 out of 30 fish tested at the two lower temperatures (6 and 10 ${}^{\circ }$C). Determining whether chronic infection or viral latency are mechanism driving persistent infections would be beneficial in future work, as latency has not been demonstrated for IHNV. However, our finding of long-term persistence within fish provides valuable information about low frequency events that could have major impacts on IHNV transmission ecology.

It important to recognize that molecular methods do not establish whether the persistent RNA is associated with infectious virus. Infectious virus was isolated from mortalities that occurred between 40 and 267 dpe indicating that viable virus was present long-term in some fraction of individuals in the 6 and 10 ${}^{\circ }$C groups. However, we were unable to confirm infectious virus by culture in randomly sampled survivors from 270 dpe, but the sample size tested was small. We did not evaluate whether this virus was transmissible to naïve fish. A recent study demonstrated that a small number of persistently infected Pacific herring can shed virus and transmit the virus to naïve fish through waterborne route [62]. The authors hypothesize that persistently infected herring may be an important means of maintaining the virus in these forage fish populations. Future work on IHNV should focus on quantifying the relationship between such persistence and among host transmission. Additionally, it has been speculated that persistent or latent infections acquired during the juvenile phase may be re-activated in adults during the stress of spawning [9], but at least one study in sockeye salmon failed to find evidence for this hypothesis [27]. Our results provide insights on the clearance time of IHNV, a crucial parameter for ongoing efforts to model IHNV transmission. Future work quantifying the probability of persistent infections and the likelihood that this persistent virus is transmitted are crucial parameters for ongoing efforts to model IHNV transmission on the landscape.

## 5. Conclusions

Environmental conditions are known to affect the dynamics of host pathogen interactions; however, it is often difficult to disentangle the effects of the environment on the host, the pathogen or the interaction. Here we show that IHNV persistence and host mortality are clearly affected by temperature, and we hypothesize that these effects are largely mediated by water temperature influencing the host immune response. We show using cell lines that increased temperatures favored faster IHNV replication. However, in vivo, the higher temperatures promoted a faster onset of mortality in IHNV exposed steelhead, but also resulted in a faster pathogen clearance and an overall lower cumulative IHNV-specific mortality over time. Increased temperature was also associated with a stronger neutralizing antibody host response at 1–3 months post-exposure with the poorest response in the colder water treatment. Thus, we suggest that clearance of IHNV infection is regulated by the effects of temperature on the host immune system. This hypothesis is supported by the higher production of neutralizing antibodies observed earlier for fish at higher water temperatures.

Overall, our results show that environmental variability can modulate host–pathogen interactions within vertebrate hosts. Because IHNV genotypes interact with a diverse community of salmonid hosts, more work is needed to understand the diversity of persistence times of different host-IHNV lineages combinations. The impact of disease on aquatic organisms is likely amplified by climate change and other human alterations to ecological systems. Therefore, studying the mechanisms driving host–pathogen interactions under different environmental conditions provides key knowledge to understand and mitigate the consequences of disease in managed wildlife.

## Figures and Tables

**Table 1:** Estimates (95% confidence intervals) of the final viral load, rate of viral load decay, and half-time (i.e., the time to which the initial value is decreased by one half) obtained from the decay model. The decay rate is in the log10 number of RNA copies/μg RNA per reaction per day and the half-time is in dpe.

Temperature	Final Viral Load (*L*)	Rate of Decay λ (VL/Day)	Half-Time (dpe)
6	1.98 (1.33, 2.62)	0.026 (0.008, 0.043)	59 (35, 189)
10	2.14 (1.75, 2.52)	0.087 (0.041, 0.134)	12 (7, 30)
15	1.59 (1.15, 2.03)	0.242 (−0.232, 0.716)	3 (NA)

**Table 2:** Magnitude of antibody production. Values are counts corresponding to the number of samples testing positive for antibodies at different serum dilutions. Counts at higher dilutions suggest a larger production of antibodies. Dilutions were Small = 1:80 and 1:160; Intermediate = 1:320 and 1:640; Large = 1:1280 and 1:2560.

		Days Post Exposure		
Temperature	Dilution	31	55	91
	Negative	1.0 (15/15)	0.73 (22/30)	0.87 (13/15)
	Small	-	0.13 (4/30)	0.13 (2/15)
6	Intermediate	-	0.10 (3/30)	-
	Large	-	0.03 (1/30)	-
	Negative	1.0 (15/15)	0.33 (10/30)	0.73(11/15)
	Small	-	0.20 (6/30)	0.20 (3/15)
10	Intermediate	-	0.26 (8/30)	0.07 (1/15)
	Large	-	0.20 (6/30)	-
	Negative	0.78 (14/18)	0.38 (11/29)	0.61 (17/28)
	Small	0.22 (4/18)	0.38 (11/29)	0.14 (4/28)
15	Intermediate	-	0.10 (3/29)	0.10 (3/28)
	Large	-	0.14 (4/29)	0.14 (4/28)

## Data Availability

Raw data and machine-readable metadata are accessible through a US Geological Survey data release: https://doi.org/10.5066/P9T4PH4Z (accessed on 3 July 2021) [63].

## Supplementary Materials

The following are available online at https://www.mdpi.com/article/10.3390/pathogens10070855/s1, Figure S1: Number of fish testing positive for IHN in different body tissues, Table S1: Akaike Information Criteria scores for 16 candidate models explaining cell culture viral kinetics. The full model (Model 1) contained the main effects of temperature, days since exposure and multiplicity of infection (MOI) treatment, as well as their interactions. The random effects were the experimental replicate and were same for the 16 models. All other models are a simpler version of Model 1, omitting different effects. * Models did not converge, Table S2: Akaike Information Criteria scores (AIC) for 5 candidate models explaining the probability of infection of live fish. The full model (Model 1) consists of the probability of infection as a function of temperature treatment, the day since exposure and their interaction. Models 2–5 are simpler versions of Model 1, each omitting an effect, Table S3: Akaike Information Criteria scores (AIC) for 16 candidate models explaining the probability of infection of different tissues in live fish. The full model (Model 1) consists of the probability of infection as a function of the main effects of temperature, the day since exposure, and the tissue, in addition to their interactions. Models 2–16 are simpler versions of Model 1, each omitting an effect.

## Abbreviations

The following abbreviations are used in this manuscript:

MDPI	Multidisciplinary Digital Publishing Institute
DOAJ	Directory of open access journals
TLA	Three letter acronym
LD	Linear dichroism
