# Supplementary material for: Temperature Variation and Host Immunity Regulate Viral Persistence in a Salmonid Host
Source: Pathogens. 2021 Jul 7;10(7):855. doi: 10.3390/pathogens10070855 (PMC8308775; doi:10.3390/pathogens10070855)
Supplement: Supplementary file 1 [file pathogens-10-00855-s001.zip › pathogens-1215733-supplementary/pathogens-1215733-supplementary.pdf]

pathogens

Article

Temperature Variation and Host Immunity Regulate Viral Persistence in a Salmonid Host

David J. Páez [1,*], Rachel L. Powers [2], Peng Jia [2,3,4], Natalia Ballesteros [2,5], Gael Kurath [2], Kerry A. Naish [1], and Maureen K. Purcell [2,*]

1 School of Aquatic and Fishery Sciences, University of Washington, Seattle, WA 98195, USA; knaish@uw.edu
2 US Geological Survey, Western Fisheries Research Center, Seattle, WA 98115, USA; rpowers@usgs.gov (R.L.P.); ajack2012@hotmail.com (P.J.); naballesterosb@gmail.com (N.B.); gkurath@usgs.gov (G.K.)
3 Shenzhen Customs, Animal & Plant Inspection and Quarantine Technology Center, Shenzhen 518045, China
4 Shenzhen Technology University, Shenzhen 518118, China
5 Department of Microbiology, University of Alabama at Birmingham, Birmingham, AL 35294, USA
* Correspondence: dpaezmc@gmail.com (D.J.P.); mpurcell@usgs.gov (M.K.P.)

check for updates

Citation: Páez, D.J.; Powers, R.L.; Jia, P.; Ballesteros, N.; Kurath, G.; Naish, K.A.; Purcell, M.K. Temperature Variation and Host Immunity Regulate Viral Persistence in a Salmonid Host. *Pathogens* **2021**, *10*, 855. https://doi.org/10.3390/pathogens10070855

Academic Editor: Lawrence S. Young

Received: 24 April 2021
Accepted: 23 June 2021
Published: 7 July 2021

Publisher's Note: MDPI stays neutral with regard to jurisdictional claims in published maps and institutional affiliations.

Copyright: © 2021 by the authors. Licensee MDPI, Basel, Switzerland. This article is an open access article distributed under the terms and conditions of the Creative Commons Attribution (CC BY) license (https://creativecommons.org/licenses/by/4.0/).

Supplementary Materials:

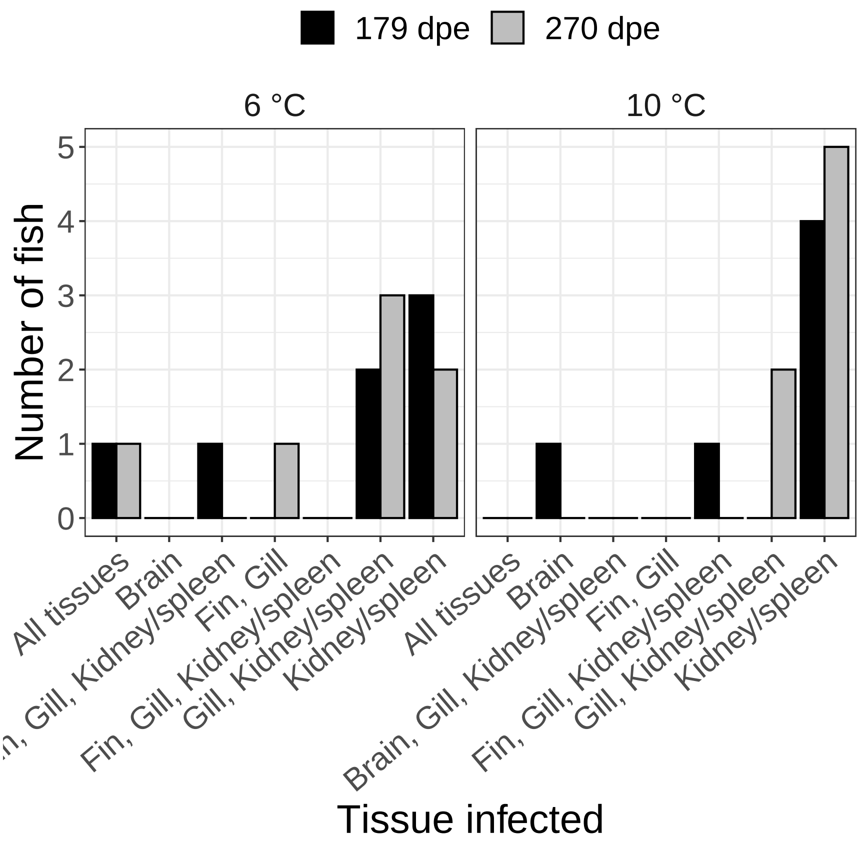

Figure S1. Number of fish testing positive for IHN in different body tissues.

Table S1: Akaike Information Criteria scores for 16 candidate models explaining cell culture viral kinetics. The full model (Model 1) contained the main effects of temperature, days since exposure and multiplicity of infection (MOI) treatment, as well as their interactions. The random effects were the experimental replicate and were same for the 16 models. All other models are a simpler version of Model 1, omitting different effects. * Models did not converge.

Model description	df	AIC	DeltaAIC	*w*AIC
Model 1, Full model	14	380.51	18.84	0.00
Model 2, Omitting interactions: triple interaction	12	368.19	6.52	0.02
Model 3, Omitting interactions: Temperature and MOI	11	369.76	8.09	0.01
Model 4, Omitting interactions: Day and MOI	10	361.67	0.00	**0.60**
Model 5, Omitting interactions: Temperature and Day, Temperature and MOI	10	459.21	97.54	0.00
Model 6, Omitting interactions: Temperature and Day, MOI and Day	8	448.27	86.60	0.00
Model 7, Omitting interactions: Temperature and MOI, Temperature and Day	9	456.07	94.40	0.00
Model 8, Omitting interactions: Temperature and MOI, Day and MOI	9	362.63	0.96	0.37
Model 9*, Omitting interactions: All double interactions	7	444.98	83.31	0.00
Model 10*, Omitting effects: MOI	6	501.30	139.63	0.00
Model 11, Omitting effects: Temperature	6	580.37	218.70	0.00
Model 12*, Omitting effects: Day	5	503.46	141.79	0.00
Model 13, Omitting effects: Temperature and MOI	5	610.67	249.00	0.00
Model 14, Omitting effects: Day and MOI	4	558.75	197.08	0.00
Model 15, Omitting effects: Temperature and Day	4	636.78	275.11	0.00
Model 16, Omitting effects: All main effects	3	665.63	303.96	0.00

Table S2: Akaike Information Criteria scores (AIC) for 5 candidate models explaining the probability of infection of live fish. The full model (Model 1) consists of the probability of infection as a function of temperature treatment, the day since exposure and their interaction. Models 2-5 are simpler versions of Model 1, each omitting an effect.

Model description	df	AIC	ΔAIC	*w*AIC
Model 1, Full model	6	503.43	0.00	**0.64**
Model 2, Omitting interactions: Temperature and Day	4	504.57	1.14	0.36
Model 3, Omitting effect: Day	3	591.12	87.69	0.00
Model 4, Omitting effect: Temperature	2	520.25	16.82	0.00
Model 5, Omitting effect: Temperature and Day	1	589.45	86.02	0.00

Table S3: Akaike Information Criteria scores (AIC) for 16 candidate models explaining the probability of infection of different tissues in live fish. The full model (Model 1) consists of the probability of infection as a function of the main effects of temperature, the day since exposure, and the tissue, in addition to their interactions. Models 2-16 are simpler versions of Model 1, each omitting an effect.

Model description	df	AIC	ΔAIC	*w*AIC
Model 1, Full model	16	127.33	9.62	0.00
Model 2, Omitting interactions: triple interaction	13	129.97	12.26	0.00
Model 3, Omitting interactions: Tissue and Temperature	10	125.11	7.40	0.01
Model 4, Omitting interactions: Day and Temperature	12	128.64	10.93	0.00
Model 5, Omitting interactions: Day and Tissue	10	125.68	7.97	0.01
Model 6, Omitting interactions: Temperature and Day	9	123.66	5.95	0.03
Model 7, Omitting interactions: Tissue and Day, Tissue and Temperature	7	120.73	3.02	0.12
Model 8, Omitting interactions: Tissue and Day, Day and Temperature	9	124.71	7.00	0.02
Model 9, Omitting effects: All double and triple interactions	6	119.71	2.00	0.20
Model 10, Omitting effects: Temperature	5	124.48	6.77	0.02
Model 11, Omitting effects: Tissue	3	160.44	42.73	0.00
Model 12, Omitting effects: Day	5	117.71	0.00	**0.54**
Model 13, Omitting effects: Temperature and Tissue	2	162.74	45.03	0.00
Model 14, Omitting effects: Temperature and Day	4	122.54	4.83	0.05
Model 15, Omitting effects: Day and Tissue	2	158.44	40.73	0.00
Model 16, Omitting all effects	1	160.78	43.07	0.00
