# Supplementary figures and images for: Temperature Variation and Host Immunity Regulate Viral Persistence in a Salmonid Host
Source: Pathogens. 2021 Jul 7;10(7):855. doi: 10.3390/pathogens10070855 (PMC8308775; doi:10.3390/pathogens10070855)

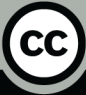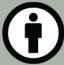

BY

Supplement: Supplementary file 1 [file pathogens-10-00855-s001.zip › pathogens-1215733-supplementary/Definitions/logo-ccby-eps-converted-to.pdf]

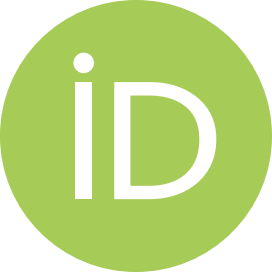

Supplement: Supplementary file 1 [file pathogens-10-00855-s001.zip › pathogens-1215733-supplementary/Definitions/logo-orcid-eps-converted-to.pdf]

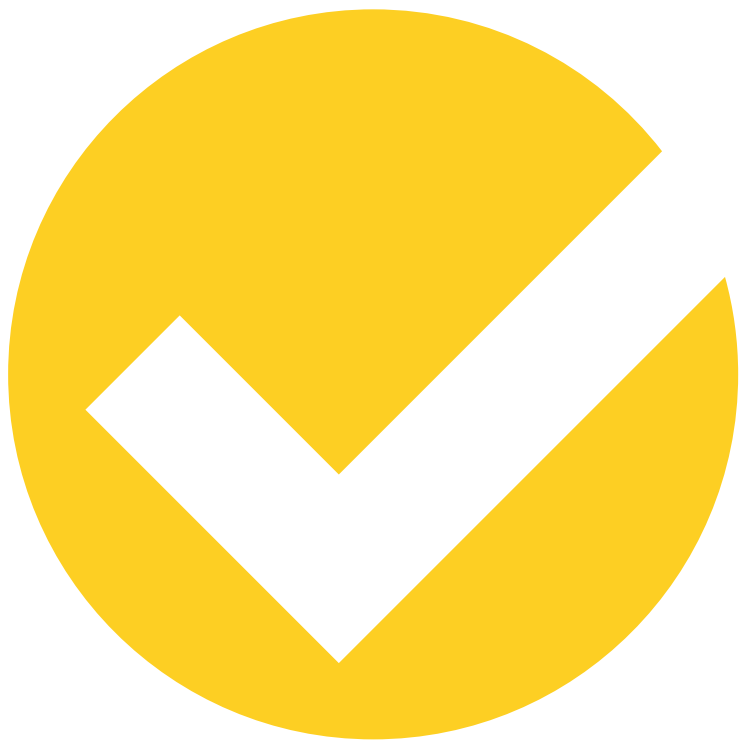

check for  
updates

Supplement: Supplementary file 1 [file pathogens-10-00855-s001.zip › pathogens-1215733-supplementary/Definitions/logo-updates.pdf]

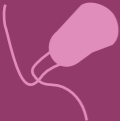

*pathogens*

Supplement: Supplementary file 1 [file pathogens-10-00855-s001.zip › pathogens-1215733-supplementary/Definitions/pathogens-logo-eps-converted-to.pdf]

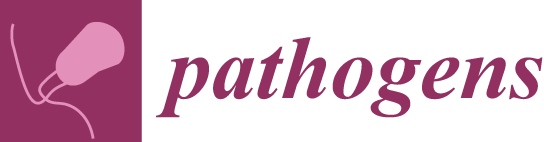

Supplement: Supplementary file 1 [file pathogens-10-00855-s001.zip › pathogens-1215733-supplementary/Definitions/pathogens-logo.png]

179 dpe 270 dpe

6 °C

10 °C

Number of fish

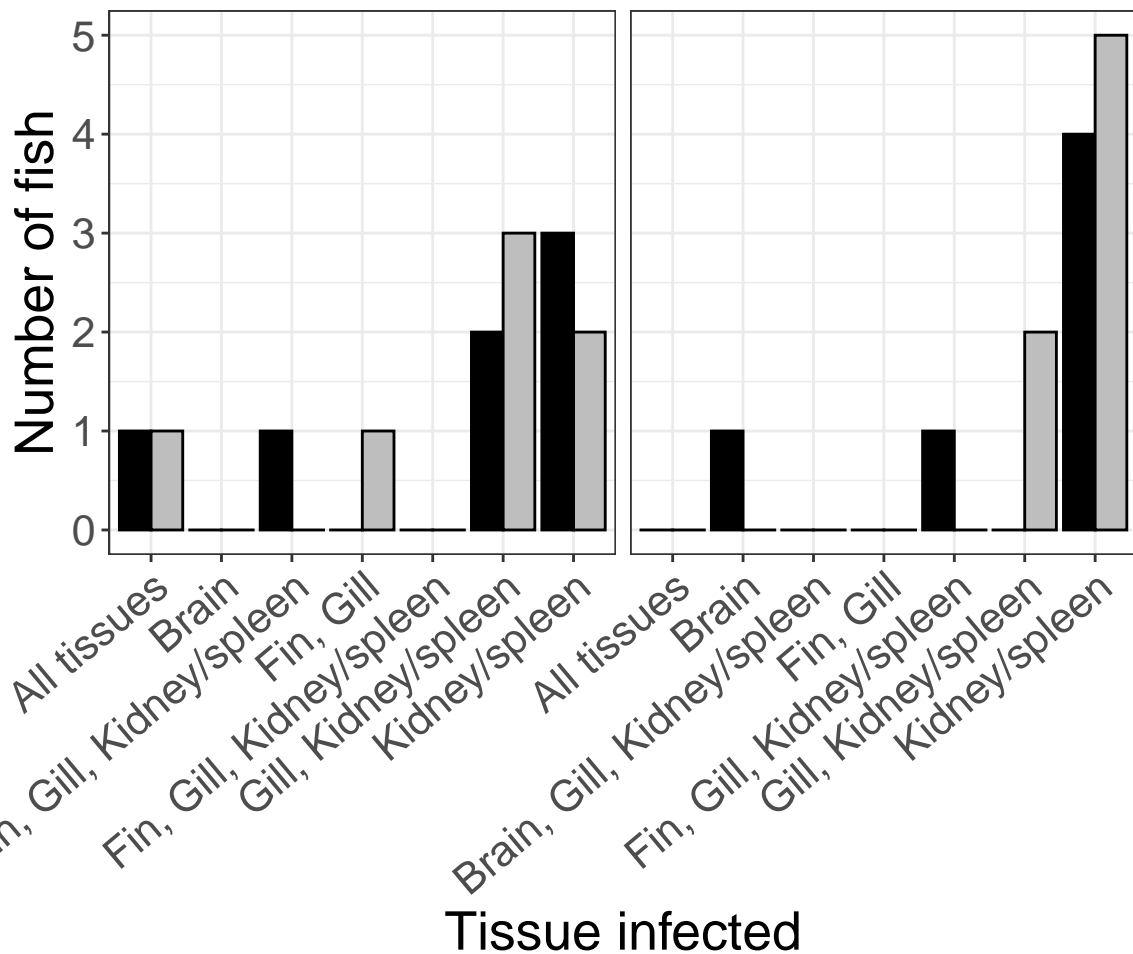

Supplement: Supplementary file 1 [file pathogens-10-00855-s001.zip › pathogens-1215733-supplementary/Tissue_supp_plot.pdf]
